# Supplementary material for: Evolution of aberrant brain‐wide spatiotemporal dynamics of resting‐state networks in a Huntington's disease mouse model
Source: Clin Transl Med. 2024 Oct 18;14(10):e70055. doi: 10.1002/ctm2.70055 (PMC11488302; doi:10.1002/ctm2.70055)
Supplement: Supplementary file 2 — Supporting information [file CTM2-14-e70055-s001.docx]

**Supplementary tables**

| Region | Genotype*Age (*p*) | Genotype (*p*) | Age (*p*) |
| --- | --- | --- | --- |
| mCPu | **<0.0001** | **<0.0001** | **<0.0001** |
| lCPu | **<0.0001** | **<0.0001** | **<0.0001** |
| PiriCtx | **<0.0001** | **<0.0001** | **<0.0001** |
| InsCtx | **<0.0001** | **<0.0001** | **<0.0001** |
| MCtx1 | **0.0375** | **0.0004** | **0.0138** |
| MCtx2 | **0.0019** | **<0.0001** | **0.0013** |
| S1Ctx | **0.0012** | **<0.0001** | **0.0003** |
| CgCtx | **<0.0001** | **<0.0001** | **<0.0001** |

**Table S1. 2B4 coverage in cells: p values of Two-way ANOVA**

| Region | Genotype*Age (*p*) | Genotype (*p*) | Age (*p*) |
| --- | --- | --- | --- |
| mCPu | 0.4684 | 0.8525 | 0.3301 |
| lCPu | 0.1866 | 0.7737 | 0.7470 |
| PiriCtx | 0.3482 | 0.7483 | 0.2048 |
| InsCtx | 0.9209 | 0.6004 | 0.1225 |
| MCtx1 | 0.2551 | 0.4891 | 0.6789 |
| MCtx2 | 0.4007 | 0.1306 | 0.7450 |
| S1Ctx | 0.2913 | 0.9165 | 0.5332 |
| CgCtx | 0.5580 | 0.6421 | 0.3777 |

**Table S2. CD13 area: p values of Two-way ANOVA**

| Region | Genotype*Age (*p*) | Genotype (*p*) | Age (*p*) |
| --- | --- | --- | --- |
| mCPu | 0.5024 | 0.8611 | 0.1534 |
| lCPu | **0.0279** | 0.6999 | 0.5962 |
| PiriCtx | 0.1109 | 0.6225 | 0.0505 |
| InsCtx | 0.6524 | 0.4104 | 0.0896 |
| MCtx1 | 0.6725 | 0.3105 | 0.8251 |
| MCtx2 | 0.2427 | **0.0262** | 0.1765 |
| S1Ctx | **0.0089** | 0.4798 | 0.1039 |
| CgCtx | 0.3180 | 0.2623 | 0.1580 |

**Table S3. CD13+ cell count: p values of Two-way ANOVA**

|  |  | Interaction | Age | Region |  |  |  |  |  |
| --- | --- | --- | --- | --- | --- | --- | --- | --- | --- |
| Two-way ANOVA | | **<0.0001** | **<0.0001** | **<0.0001** |  |  |  |  |  |
|  |  | **3 months** | | **6 months** | | **8 months** | | **12 months** | |
| Post-hoc pairs | | orig p value | FDR (p<0.05) | orig p value | FDR (p<0.05) | orig p value | FDR (p<0.05) | orig p value | FDR (p<0.05) |
| mCPu vs. lCPu | | 0.3571 | 0.9994 | 0.0207 | 0.0528 (#) | 0.054 | 0.1164 | 0.5203 | 0.711 |
| mCPu vs. CgCtx | | 0.221 | 0.9994 | 0.0001 | **0.0015 (**)** | <0,0001 | **<0,0001 (****)** | <0,0001 | **<0,0001 (****)** |
| mCPu vs. S1Ctx | | 0.2287 | 0.9994 | 0.6871 | 0.7374 | <0,0001 | **<0,0001 (****)** | <0,0001 | **<0,0001 (****)** |
| mCPu vs. InsCtx | | 0.2207 | 0.9994 | 0.0001 | **0.0015 (**)** | <0,0001 | **0.0003 (***)** | <0,0001 | **<0,0001 (****)** |
| mCPu vs. PiriCtx | | 0.2973 | 0.9994 | 0.0008 | **0.0046 (**)** | <0,0001 | **<0,0001 (****)** | <0,0001 | **0.0002 (***)** |
| mCPu vs. MCtx 1 | | 0.4763 | 0.9994 | 0.0052 | **0.0209 (*)** | <0,0001 | **<0,0001 (****)** | <0,0001 | **<0,0001 (****)** |
| mCPu vs. MCtx 2 | | 0.6592 | 0.9994 | 0.1949 | 0.3211 | <0,0001 | **<0,0001 (****)** | <0,0001 | **<0,0001 (****)** |
| lCPu vs. CgCtx | | 0.8472 | 0.9994 | 0.1185 | 0.2212 | 0.0076 | **0.0217 (*)** | <0,0001 | **<0,0001 (****)** |
| lCPu vs. S1Ctx | | 0.8622 | 0.9994 | 0.0527 | 0.1135 | 0.0046 | **0.0182 (*)** | <0,0001 | **<0,0001 (****)** |
| lCPu vs. InsCtx | | 0.8467 | 0.9994 | 0.1485 | 0.26 | 0.0198 | **0.0461 (*)** | <0,0001 | **<0,0001 (****)** |
| lCPu vs. PiriCtx | | 0.9504 | 0.9994 | 0.3023 | 0.4232 | 0.0077 | **0.0217 (*)** | <0,0001 | **<0,0001 (****)** |
| lCPu vs. MCtx 1 | | 0.8025 | 0.9994 | 0.6343 | 0.7104 | 0.0089 | **0.0227 (*)** | <0,0001 | **<0,0001 (****)** |
| lCPu vs. MCtx 2 | | 0.5862 | 0.9994 | 0.2746 | 0.4066 | 0.0052 | **0.0182 (*)** | <0,0001 | **<0,0001 (****)** |
| CgCtx vs. S1Ctx | | 0.983 | 0.9994 | 0.0004 | **0.0034 (**)** | 0.8724 | 0.9708 | 0.4329 | 0.6734 |
| CgCtx vs. InsCtx | | 0.9994 | 0.9994 | 0.8514 | 0.8514 | 0.7378 | 0.9708 | 0.5333 | 0.711 |
| CgCtx vs. PiriCtx | | 0.8914 | 0.9994 | 0.5934 | 0.6923 | 0.9956 | 0.9956 | 0.0708 | 0.1524 |
| CgCtx vs. MCtx 1 | | 0.6296 | 0.9994 | 0.2759 | 0.4066 | 0.8584 | 0.9708 | 0.7384 | 0.8615 |
| CgCtx vs. MCtx 2 | | 0.4103 | 0.9994 | 0.007 | **0.0244 (*)** | 0.9014 | 0.9708 | 0.4787 | 0.7055 |
| S1Ctx vs. InsCtx | | 0.9824 | 0.9994 | 0.0005 | **0.0034 (**)** | 0.6205 | 0.9708 | 0.8716 | 0.9386 |
| S1Ctx vs. PiriCtx | | 0.9075 | 0.9994 | 0.0029 | **0.0133 (*)** | 0.868 | 0.9708 | 0.3029 | 0.4989 |
| S1Ctx vs. MCtx 1 | | 0.6441 | 0.9994 | 0.0154 | **0.0432 (*)** | 0.7292 | 0.9708 | 0.6521 | 0.8299 |
| S1Ctx vs. MCtx 2 | | 0.4225 | 0.9994 | 0.3704 | 0.4714 | 0.9707 | 0.9956 | 0.9394 | 0.9394 |
| InsCtx vs. PiriCtx | | 0.8909 | 0.9994 | 0.7111 | 0.7374 | 0.742 | 0.9708 | 0.2337 | 0.4363 |
| InsCtx vs. MCtx 1 | | 0.6291 | 0.9994 | 0.3414 | 0.4552 | 0.8641 | 0.9708 | 0.7723 | 0.865 |
| InsCtx vs. MCtx 2 | | 0.4099 | 0.9994 | 0.0084 | **0.026 (*)** | 0.6466 | 0.9708 | 0.9318 | 0.9394 |
| PiriCtx vs. MCtx 1 | | 0.7405 | 0.9994 | 0.5773 | 0.6923 | 0.8629 | 0.9708 | 0.1395 | 0.2791 |
| PiriCtx vs. MCtx 2 | | 0.5164 | 0.9994 | 0.031 | 0.0724 (#) | 0.8971 | 0.9708 | 0.2688 | 0.4704 |
| MCtx 1 vs. MCtx 2 | | 0.7616 | 0.9994 | 0.113 | 0.2212 | 0.7583 | 0.9708 | 0.7077 | 0.8615 |

**Table S4. 2B4 coverage in CD13+ cells: p-values of Two-way ANOVA and post-hoc comparisons**

|  |  | Interaction | Age | Region |  |  |  |  |  |
| --- | --- | --- | --- | --- | --- | --- | --- | --- | --- |
| Two-way ANOVA | | **0.0008** | **<0.0001** | **<0.0001** |  |  |  |  |  |
|  |  | **3 months** | | **6 months** | | **8 months** | | **12 months** | |
|  | | orig p value | FDR (p<0.05) | orig p value | FDR (p<0.05) | orig p value | FDR (p<0.05) | orig p value | FDR (p<0.05) |
| mCPu vs. lCPu | | 0.9944 | >0,9999 | 0.0742 | 0.2828 | 0.0434 | 0.0794 (#) | 0.001 | **0.0039 (**)** |
| mCPu vs. CgCtx | | 0.9978 | >0,9999 | 0.0009 | **0.0241 (*)** | <0,0001 | **0.0004 (***)** | 0.0009 | **0.0039 (**)** |
| mCPu vs. S1Ctx | | 0.9942 | >0,9999 | 0.1422 | 0.3369 | 0.0004 | **0.0011 (***)** | 0.0007 | **0.0039 (**)** |
| mCPu vs. InsCtx | | 0.9944 | >0,9999 | 0.0089 | 0.0623 (#) | 0.0001 | **0.0004 (***)** | 0.0019 | **0.0065 (**)** |
| mCPu vs. PiriCtx | | 0.9944 | >0,9999 | 0.0059 | 0.055 (#) | 0.8659 | 0.8723 | 0.6053 | 0.7369 |
| mCPu vs. M1Ctx | | 0.9567 | >0,9999 | 0.136 | 0.3369 | <0,0001 | **0.0003 (***)** | 0.1399 | 0.2449 |
| mCPu vs. M2Ctx | | 0.9942 | >0,9999 | 0.0025 | **0.0347 (*)** | <0,0001 | **0.0003 (***)** | 0.0124 | **0.0347 (*)** |
| lCPu vs. CgCtx | | 0.9963 | >0,9999 | 0.1499 | 0.3369 | 0.0392 | 0.0785 (#) | 0.5445 | 0.693 |
| lCPu vs. S1Ctx | | >0,9999 | >0,9999 | 0.7461 | 0.8557 | 0.0822 | 0.1355 | 0.8369 | 0.8918 |
| lCPu vs. InsCtx | | >0,9999 | >0,9999 | 0.3917 | 0.5773 | 0.034 | 0.0733 (#) | 0.86 | 0.8918 |
| lCPu vs. PiriCtx | | >0,9999 | >0,9999 | 0.3172 | 0.4934 | 0.0454 | 0.0794 (#) | 0.0005 | **0.0039 (**)** |
| lCPu vs. M1Ctx | | 0.9509 | >0,9999 | 0.764 | 0.8557 | 0.0229 | 0.0534 (#) | 0.0427 | 0.1086 |
| lCPu vs. M2Ctx | | 0.9883 | >0,9999 | 0.1564 | 0.3369 | 0.0179 | **0.0455 (*)** | 0.191 | 0.3146 |
| CgCtx vs. S1Ctx | | 0.9962 | >0,9999 | 0.0737 | 0.2828 | 0.8126 | 0.8723 | 0.6741 | 0.7697 |
| CgCtx vs. InsCtx | | 0.9963 | >0,9999 | 0.6125 | 0.7898 | 0.8607 | 0.8723 | 0.6872 | 0.7697 |
| CgCtx vs. PiriCtx | | 0.9963 | >0,9999 | 0.7277 | 0.8557 | <0,0001 | **0.0003 (***)** | 0.0005 | **0.0039 (**)** |
| CgCtx vs. M1Ctx | | 0.9523 | >0,9999 | 0.0779 | 0.2828 | 0.7301 | 0.8723 | 0.0806 | 0.1505 |
| CgCtx vs. M2Ctx | | 0.9916 | >0,9999 | 0.8281 | 0.8918 | 0.5513 | 0.8125 | 0.3879 | 0.5173 |
| S1Ctx vs. InsCtx | | >0,9999 | >0,9999 | 0.2388 | 0.4088 | 0.6955 | 0.8723 | 0.9862 | 0.9862 |
| S1Ctx vs. PiriCtx | | >0,9999 | >0,9999 | 0.1864 | 0.3627 | 0.0003 | 0.0008 (***) | 0.0004 | **0.0039 (**)** |
| S1Ctx vs. M1Ctx | | 0.9485 | >0,9999 | 0.9812 | 0.9812 | 0.581 | 0.8134 | 0.0482 | 0.1125 |
| S1Ctx vs. M2Ctx | | 0.9877 | >0,9999 | 0.0869 | 0.2828 | 0.437 | 0.6797 | 0.2339 | 0.3638 |
| InsCtx vs. PiriCtx | | >0,9999 | >0,9999 | 0.885 | 0.9178 | <0,0001 | **0.0003 (***)** | 0.001 | **0.0039 (**)** |
| InsCtx vs. M1Ctx | | 0.9509 | >0,9999 | 0.2482 | 0.4088 | 0.8723 | 0.8723 | 0.0666 | 0.1366 |
| InsCtx vs. M2Ctx | | 0.9883 | >0,9999 | 0.5295 | 0.7413 | 0.6776 | 0.8723 | 0.2688 | 0.3961 |
| PiriCtx vs. M1Ctx | | 0.9509 | >0,9999 | 0.1943 | 0.3627 | <0,0001 | **0.0003 (***)** | 0.0683 | 0.1366 |
| PiriCtx vs. M2Ctx | | 0.9883 | >0,9999 | 0.6206 | 0.7898 | <0,0001 | **0.0003 (***)** | 0.0062 | **0.0192 (*)** |
| M1Ctx vs. M2Ctx | | 0.9608 | >0,9999 | 0.0909 | 0.2828 | 0.7894 | 0.8723 | 0.351 | 0.4914 |

**Table S5. 2B4 coverage in GFAP+ cells: p-values of Two-way ANOVA and post-hoc comparisons**

| Region | Genotype*Age (*p*) | Genotype (*p*) | Age (*p*) |
| --- | --- | --- | --- |
| mCPu | 0.2914 | 0.9409 | 0.3845 |
| lCPu | **0.0389** | 0.1804 | 0.0774 |
| PiriCtx | 0.4688 | 0.4257 | 0.0777 |
| InsCtx | 0.4797 | **0.0006** | **<0.0001** |
| MCtx1 | 0.0889 | 0.2428 | 0.9958 |
| MCtx2 | **0.0107** | 0.6881 | 0.6278 |
| S1Ctx | 0.1390 | 0.0630 | **0.0064** |
| CgCtx | 0.3117 | 0.2883 | 0.0845 |

**Table S6. GFAP area: p values of Two-way ANOVA**

| Region | Genotype*Age (*p*) | Genotype (*p*) | Age (*p*) |
| --- | --- | --- | --- |
| mCPu | 0.1968 | 0.7400 | 0.4816 |
| lCPu | 0.2142 | 0.0709 | 0.0516 |
| PiriCtx | 0.7811 | 0.3715 | **0.0242** |
| InsCtx | 0.6989 | **0.0110** | **0.0021** |
| MCtx1 | 0.2053 | 0.3117 | 0.1223 |
| MCtx2 | 0.0855 | 0.8228 | 0.3318 |
| S1Ctx | 0.8360 | 0.8551 | **0.0022** |
| CgCtx | 0.4123 | 0.0737 | 0.1672 |

**Table S7. GFAP+ cell count: p values of Two-way ANOVA**

| Region | Genotype*Age (*p*) | Genotype (*p*) | Age (*p*) |
| --- | --- | --- | --- |
| mCPu | 0.2270 | 0.4123 | **0.0126** |
| lCPu | **<0.0001** | **<0.0001** | **<0.0001** |
| PiriCtx | **<0.0001** | **<0.0001** | **<0.0001** |
| InsCtx | **0.0002** | **<0.0001** | **0.0001** |
| MCtx1 | 0.2228 | 0.0940 | **0.0007** |
| MCtx2 | 0.1909 | 0.0790 | **0.0001** |
| S1Ctx | **0.0009** | **0.0007** | **<0.0001** |
| CgCtx | **0.0170** | **0.0005** | **<0.0001** |

**Table S8. VEGF mean intensity : p values of Two-way ANOVA**
